# Inferring Regulatory Networks from Expression Data Using Tree-Based Methods

**DOI:** 10.1371/journal.pone.0012776

**Published:** 2010-09-28

**Authors:** Vân Anh Huynh-Thu, Alexandre Irrthum, Louis Wehenkel, Pierre Geurts

**Affiliations:** 1 Department of Electrical Engineering and Computer Science, Systems and Modeling, University of Liège, Liège, Belgium; 2 GIGA-Research, Bioinformatics and Modeling, University of Liège, Liège, Belgium; Center for Genomic Regulation, Spain

## Abstract

One of the pressing open problems of computational systems biology is the elucidation of the topology of genetic regulatory networks (GRNs) using high throughput genomic data, in particular microarray gene expression data. The Dialogue for Reverse Engineering Assessments and Methods (DREAM) challenge aims to evaluate the success of GRN inference algorithms on benchmarks of simulated data. In this article, we present GENIE3, a new algorithm for the inference of GRNs that was best performer in the DREAM4 *In Silico Multifactorial* challenge. GENIE3 decomposes the prediction of a regulatory network between *p* genes into *p* different regression problems. In each of the regression problems, the expression pattern of one of the genes (target gene) is predicted from the expression patterns of all the other genes (input genes), using tree-based ensemble methods Random Forests or Extra-Trees. The importance of an input gene in the prediction of the target gene expression pattern is taken as an indication of a putative regulatory link. Putative regulatory links are then aggregated over all genes to provide a ranking of interactions from which the whole network is reconstructed. In addition to performing well on the DREAM4 *In Silico Multifactorial* challenge simulated data, we show that GENIE3 compares favorably with existing algorithms to decipher the genetic regulatory network of *Escherichia coli*. It doesn't make any assumption about the nature of gene regulation, can deal with combinatorial and non-linear interactions, produces directed GRNs, and is fast and scalable. In conclusion, we propose a new algorithm for GRN inference that performs well on both synthetic and real gene expression data. The algorithm, based on feature selection with tree-based ensemble methods, is simple and generic, making it adaptable to other types of genomic data and interactions.

## Introduction

Genetic regulatory networks (GRNs) [1] are central to all biological organisms, and their deciphering is crucial to understand the development, functioning and pathology of these organisms. Once a remote theoretical possibility, this deciphering is now made possible by advances in genomics, most notably high-throughput profiling of gene expression patterns with DNA microarrays. These advances have prompted the development of a plethora of models of GRNs and algorithms to reverse-engineer them from expression data [2]–[5].

The simplest models of genetic regulatory networks are based on Boolean logic. Because of their simplicity, these Boolean network models have provided high-level insights into the design principles and emerging properties of GRNs [6]. At the other end of the complexity spectrum are physical models mimicking the biological mechanisms at play, including promoter recognition, mRNA transcription and protein translation. These models, typically based on systems of ordinary or stochastic differential equations, can generate realistic behavior [7]. One of their main drawbacks is that they have high-dimensional parameter spaces, and thus a large number of experimental data are needed for their identification. Nevertheless, hybrid methods involving ordinary differential equations have shown good performances on real-life genome-wide GRN inference [8].

Models based on the statistical analysis of dependencies between expression patterns have an intermediate complexity, and have already been successfully applied to the inference of large GRNs. Early models used correlation coefficients between expression patterns of all pairs of genes to infer “coexpression networks” [9]. However, correlation coefficients fail to capture more complex statistical dependencies (e.g. non-linear ones) between expression patterns, and thus more general measures of dependency based on mutual information (MI), have been proposed. The simplest model based on this measure, the “relevance network”, computes MI between all pairs of genes and infers the presence of a regulatory interaction when MI is larger than a given threshold [10]. Various refinements have been proposed to try to discriminate between direct and indirect interactions in relevance networks. The CLR algorithm [11] modifies the MI score based on the empirical distribution of all MI scores. The ARACNE algorithm [12] filters out indirect interactions from triplets of genes with the Data Processing Inequality [13]. Finally, MRNET [14] uses an iterative feature selection method based on a maximum relevance/minimum redundancy criterion.

Probabilistic graphical models have been widely used to model GRNs [15]. With respect to correlation or mutual information based approaches, these methods are potentially able to model higher-order dependencies between the expression patterns of genes. Among these methods, Bayesian networks have been used since the advent of microarray technologies for GRN modeling and inference [16]. A Bayesian network represents conditional dependencies between random variables with a directed acyclic graph. Learning the structure of a Bayesian network is a non trivial problem, both from a theoretical and computational point of view, and several sophisticated heuristics have been proposed in the context of GRN inference [17], [18]. One limitation of Bayesian networks for GRN inference is that these models do not allow the presence of cycles (feedback loops). While this limitation is partially circumvented by dynamic Bayesian networks [17], [19], these models can only be learned from time-series expression data. Another family of probabilistic models that gained interest recently for GRN inference are Gaussian graphical models. These methods assume that gene expression values are jointly Gaussian distributed and represent conditional dependencies between genes by an undirected graph. The estimation of this graph for high-dimensional data is difficult but several robust solutions have been proposed in the literature [20]–[23]. Although often very effective, the main limitations of these methods is of course the Gaussianity assumption, which also implies linear dependencies between variables, and the undirected nature of the inferred regulatory links (although some heuristics have been proposed to direct them [24]).

Within this context, this article presents GENIE3 (for “GEne Network Inference with Ensemble of trees”), a new GRN inference method based on variable selection with ensembles of regression trees. This method was best performer in the DREAM4 *In Silico Multifactorial* challenge [25]. Its main features with respect to existing techniques is that it makes very few assumptions about the nature of the relationships between the variables (which can thus be non-linear) and can potentially capture high-order conditional dependencies between expression patterns. It also produces a *directed* graph of regulatory interactions and naturally allows for the presence of feedback loops in the network. At the same time, it remains intuitive, computationally tractable, and easy to implement. In addition to its good performance on the synthetic data of the DREAM4 challenge, we show that GENIE3 compares favorably with existing algorithms to decipher the genetic regulatory network of *Escherichia coli*.

## Methods

### Problem Definition

We address the problem of recovering regulatory networks from gene expression data. The targeted networks are directed graphs with *p* nodes, where each node represents a gene, and an edge directed from one gene *i* to another gene *j* indicates that gene *i* (directly) regulates the expression of gene *j*. We only consider unsigned edges; when gene *i* is connected to gene *j*, the former can be either an activator or a repressor of the latter.

The goal of (unsupervised) gene regulatory network inference is to recover the network solely from measurements of the expression of the genes in various conditions. Given the dynamic and combinatorial nature of genetic regulation, measurements of different kinds can be obtained, including steady-state expression profiles resulting from the systematic knockout or knockdown of genes or time series measurements resulting from random perturbations. In this paper, we focus on multifactorial perturbation data as generated for the DREAM4 *In Silico Size 100 Multifactorial* subchallenge. Multifactorial expression data are static steady-state measurements obtained by (slightly) perturbing all genes simultaneously. Multifactorial data might correspond for example to expression profiles obtained from different patients or biological replicates. Such data are easier and less expensive to obtain than knockout/knockdown or time series data and are thus more common in practice. They are however also less informative for the prediction of edge directionality [3], [26], [27] and therefore make the regulatory network inference task more challenging.

In what follows, we define a (multifactorial) learning sample from which to infer the network as a sample of *N* measurements:
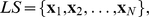
where 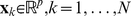 is a vector of expression values of all *p* genes in the *k*th experiment:
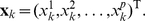



From this learning sample, the goal of network inference algorithms is to make a prediction of the underlying regulatory links between genes. Most network inference algorithms work first by providing a ranking of the potential regulatory links from the most to the less significant. A practical network prediction is then obtained by setting a threshold on this ranking. In this paper, we focus only on the first task, which is also targeted by the evaluation procedure of the DREAM4 challenge. The question of the choice of an optimal confidence threshold, although important, will be left open.

A network inference algorithm is thus defined in this paper as a procedure that exploits a *LS* to assign weights 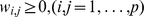 to putative regulatory links from any gene *i* to any gene *j*, with the aim of yielding large values for weights which correspond to actual regulatory interactions.

### Network Inference with Tree-based Methods

The basic idea of our procedure is to decompose the problem of recovering a network involving *p* genes into *p* different subproblems, where each of these subproblems consists in identifying the regulators of one of the genes of the network. Exploiting expression data, the identification of the regulatory genes for a given target gene is defined as determining the subset of genes whose expression directly influences or is predictive of the expression of the target gene. Within the framework of supervised learning, this problem is equivalent to a feature selection problem. In this context, our solution will exploit the embedded feature ranking mechanism of tree-based ensemble methods.

We first describe our procedure to solve the network inference problem using feature selection techniques and then specialize it to the case of tree-based ensemble methods.

### Network Inference as a Feature Selection Problem

Our method makes the assumption that the expression of each gene in a given condition is a function of the expression of the other genes in the network (plus some random noise). Denoting by 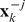 the vector containing the expression values in the *k*th experiment of all genes except gene *j*:
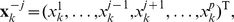
we assume that we can write:
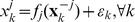
(1)where 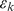 is a random noise with zero mean (conditionally to 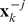). We further make the assumption that the function 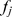 only exploits the expression in 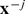 of the genes that are direct regulators of gene *j*, i.e. genes that are directly connected to gene *j* in the targeted network. Recovering the regulatory links pointing to gene *j* thus amounts at finding those genes whose expression is predictive of the expression of the target gene. In machine learning terminology, this can be considered as a feature selection problem (in regression) for which many solutions exist [28]. We assume here the use of a feature ranking technique that, instead of directly returning a feature subset, yields a ranking of the features from the most relevant to the less relevant for predicting the output.

**Figure 1:**
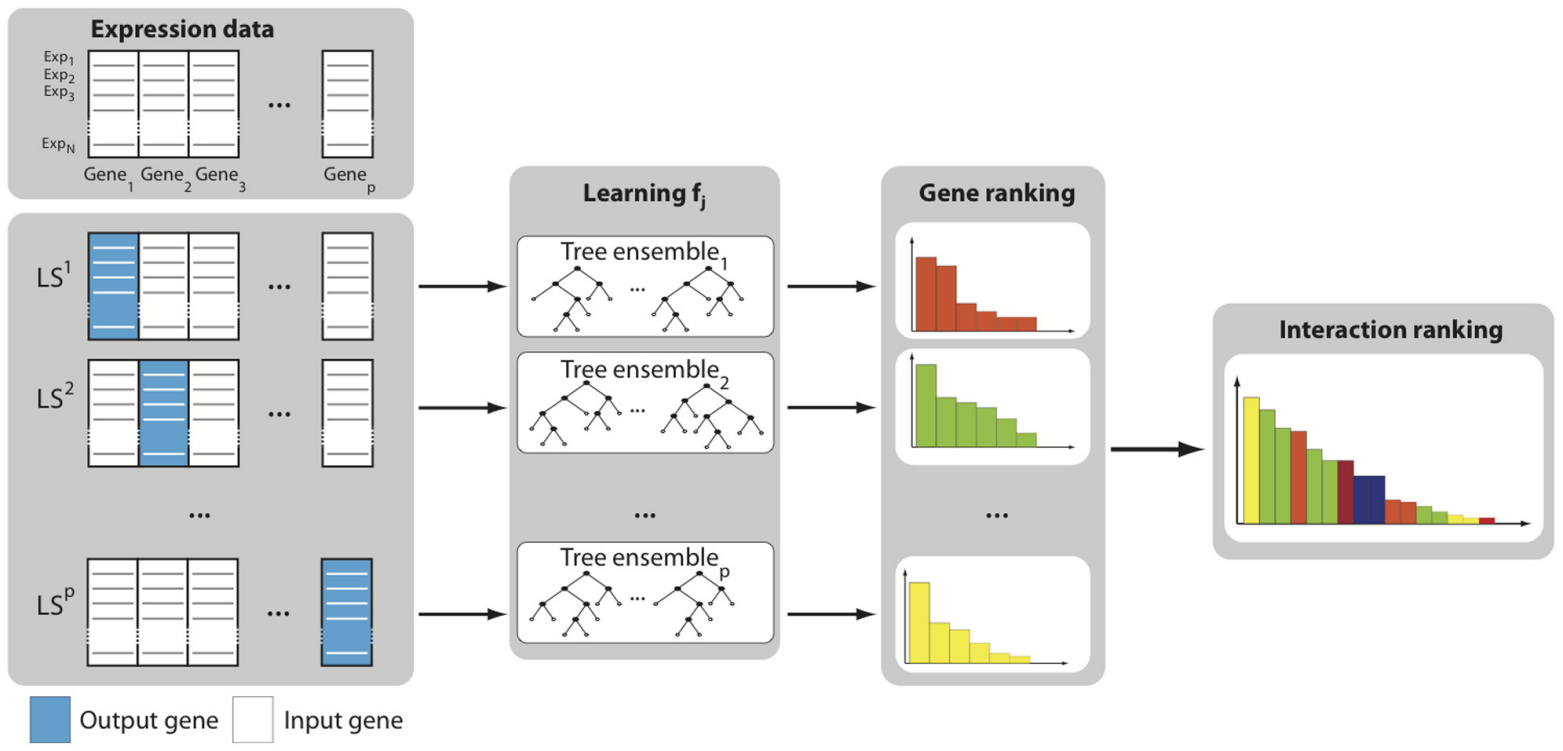
GENIE3 procedure. For each gene 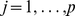, a learning sample 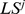 is generated with expression levels of *j* as output values and expression levels of all other genes as input values. A function 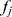 is learned from 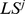 and a local ranking of all genes except *j* is computed. The *p* local rankings are then aggregated to get a global ranking of all regulatory links.

The proposed network inference procedure is illustrated in Figure 1 and works as follows:

- For *j* = 1 to *p*:
  - Generate the learning sample of input-output pairs for gene *j*:
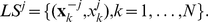

  - Use a feature selection technique on 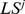 to compute confidence levels 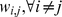, for all genes except gene *j* itself.
- Aggregate the *p* individual gene rankings to get a global ranking of all regulatory links.

Note that depending of the interpretation of the weights 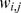, their aggregation to a get a global ranking of regulatory links is not trivial. We will see in the context of tree-based methods that it requires to normalize each expression vector appropriately.

### Gene Ranking with Tree-based Methods

The nature of the problem and the proposed solution put some constraints on candidate feature selection techniques. The nature of the functions 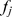 is unknown but they are expected to involve the expression of several genes (combinatorial regulation) and to be non-linear. The number of input features in each of these problems is typically much greater than the number of observations. Computationally, since the identification of a network involving *p* genes requires to rerun the algorithm *p* times, it is also of interest for this algorithm to be fast and to require as few manual tuning as possible. Tree-based ensemble methods are good candidates for that purpose. These methods do not make any assumption about the nature of the target function, can potentially deal with interacting features and non-linearity. They work well in the presence of a large number of features, are fast to compute, scalable, and essentially parameter-free (see [29] for a review).

We first briefly describe these methods and their built-in feature ranking mechanism and then discuss their use in the context of the network inference procedure described in the previous section.

### Tree-based Ensemble Methods

Each subproblem, defined by a learning sample 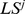, is a supervised (non-parametric) regression problem. Using square error loss, each problem amounts at finding a function 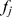 that minimizes the following error:
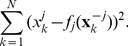
(2)Regression trees [30] solve this problem by developing tree structured models. The basic idea of this method is to recursively split the learning sample with binary tests based each on one input variable (selected in 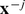), trying to reduce as much as possible the variance of the output variable (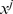) in the resulting subsets of samples. Candidate splits for numerical variables typically compare the input variable values with a threshold which is determined during the tree growing.

Single trees are usually very much improved by ensemble methods, which average the predictions of several trees. In our network inference procedure, we compare two tree-based ensemble methods based on randomization, namely Random Forests [31] and Extra-Trees [32]. In a Random Forests ensemble, each tree is built on a bootstrap sample from the original learning sample and, at each test node, *K* attributes are selected at random among all candidate attributes before determining the best split. In the Extra-Trees method on the other hand, each tree is built from the original learning sample and at each test node, the best split is determined among *K* random splits, each determined by randomly selecting one input (without replacement) and a threshold. For these two methods, we will grow ensembles of 1000 trees and we will consider two values of the main parameter of these methods: 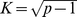 and 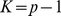, where 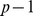 is the number of inputs, equal to the number of potential regulators of each gene. Empirical validations in [32] have shown that these two values of *K* were near-optimal in terms of predictive accuracy on several problems. Note however that we do not exclude that better results could be obtained with other settings of *K*.

### Variable Importance Measure

One of the most interesting characteristics of tree-based methods is that it is possible to compute from a tree a variable importance measure that allows to rank the input features according to their relevance for predicting the output. Several variable importance measures have been proposed in the literature for tree-based methods. In our experiment, we consider a measure which at each test node 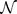 computes the total reduction of the variance of the output variable due to the split, defined by [30]:
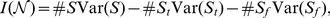
(3)where *S* denotes the set of samples that reach node 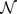, 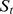 (resp. 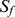) denotes its subset for which the test is true (resp. false), Var(.) is the variance of the output variable in a subset, and # denotes the cardinality of a set of samples. For a single tree, the overall importance of one variable is then computed by summing the *I* values of all tree nodes where this variable is used to split. Those attributes that are not selected at all obtain a zero value of their importance, and those that are selected close to the root node of the tree typically obtain high scores. Attribute importance measures can be easily extended to ensembles, simply by averaging importance scores over all trees in the ensemble. The resulting importance measure is then even more reliable because of the variance reduction effect resulting from this averaging.

Breiman [31] proposed an alternative measure that computes the average reduction of the tree accuracy on out-of-bag samples (i.e. training objects that are not present in the bootstrap sample used to build each tree) when the values of the corresponding variable are randomly permuted. While this procedure has some advantages with respect to the variance reduction based measure of (3) [33], it gives in most practical applications very similar results but is much more computationally demanding. Furthermore, it does not extend to methods like the Extra-Trees method which do not consider bootstrap sampling.

### Regulatory Link Ranking

Each tree-based model yields a separate ranking of the genes as potential regulators of a target gene in the form of weights 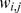 computed as sums of total variance reductions in the form (3). The sum of the importances of all variables for a tree is equal to the total variance of the output variable explained by the tree, which in the case of unpruned trees (as they are in the case of Random Forests and Extra-Trees ensembles) is usually very close to the initial total variance of the output:
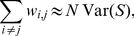
(4)where *S* is the learning sample from which the tree was built (i.e. 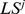 for the Extra-Trees method and a bootstrap sample for the Random Forests method) and where Var(*S*) is the variance of the target gene estimated in the corresponding learning sample. As a consequence, if we trivially order the regulatory links according to the weights 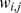, this is likely to introduce a positive bias for regulatory links towards the more highly variable genes. To avoid this bias, we have first normalized the gene expressions so that they all have a unit variance in the training set, before applying the tree-based ensemble methods. This normalization indeed implies that the different weights inferred from different models predicting the different gene expressions are comparable.

### Computational Complexity

The computational complexity of the Random Forests and Extra-Trees algorithms is on the order of 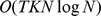, where *T* is the number of trees, *N* is the learning sample size and *K* is the main parameter of the two tree-based methods. GENIE3's complexity is thus on the order of 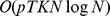 since it requires to build an ensemble of trees for each of the *p* genes. The complexity of the whole procedure is thus log linear with respect to the number of measurements and, at worst, quadratic with respect to the number of genes (when 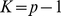).

To fix ideas, with our MatLab implementation of GENIE3, it takes 6.5 minutes to infer the five networks of the DREAM4 challenge and 7 hours to infer the *E. coli* network (with known transcription factors), in both cases with Random Forests and 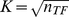, where 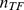 is the number of potential regulators (see later for the details of these experiments). These computing times where measured on a 16GB RAM, Intel L5420 2.50 GHz computer.

Note that, if needed, the algorithm can be easily parallelized as the *p* feature selection problems, as well as the different trees in an ensemble, are independent of each other.

## Results

### Datasets

We report below two series of experiments: first on the DREAM4 *In Silico Multifactorial* challenge and then on the *Escherichia coli* regulatory network.

### DREAM4 Datasets

The DREAM (for “Dialogue for Reverse Engineering Assessments and Methods”) initiative organizes an annual reverse engineering competition called the DREAM challenge [34]–[37]. We report here our results on the DREAM4 edition of this competition, where one challenge concerned *in silico* regulatory network inference [25]. This challenge was divided into three subchallenges, called *In Silico Size 10*, *In Silico Size 100*, and *In Silico Size 100 Multifactorial*. We only report here our result of this last subchallenge.

The goal of the *In Silico Size 100 Multifactorial* subchallenge was to infer five networks of *p* = 100 genes each from multifactorial perturbation data. Multifactorial data are defined as static steady-state expression profiles resulting from slight perturbations of all genes simultaneously.

All networks and data were generated with GeneNetWeaver (GNW) version 2.0 [38]. Network topologies were obtained by extracting subnetworks from transcriptional regulatory networks of *E. coli* and *S. cerevisiae*. The subnetwork extraction method was adapted to preferentially include parts of the network with cycles but direct self-interactions were removed. The dynamics of the networks were simulated using a detailed kinetic model of gene regulation. Noise was added both in the dynamics of the networks and on the measurement of expression data. Multifactorial perturbations were simulated by slightly increasing or decreasing the basal activation of all genes of the network simultaneously by different random amounts. In total, the number of expression conditions *N* for each network was set to 100.

### *Escherichia coli* Dataset

In addition, we carried out experiments with our method on the inference of the regulatory network of *Escherichia coli*, which has been used by several authors as a benchmark.

The dataset of expression profiles we used was retrieved from the Many Microbe Microarrays (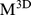) database [39] (version 4 build 6). It contains 907 *E. coli* microarray expression profiles of 4297 genes collected from different experiments at steady-state level. To validate the network predictions we used 3433 experimentally confirmed regulatory interactions among 1471 genes that have been curated in RegulonDB version 6.4 [40].

### Performance Metrics

Our algorithm provides a ranking of the regulatory links from the most confident to the less confident. To evaluate such a ranking independently of the choice of a specific threshold, we used both precision-recall (PR) curve and receiver operating characteristic (ROC) curve. The former plots for varying thresholds on the importance scores the proportion of true positives among all predictions (precision) versus the percentage of true positives that are retrieved (recall), whereas a ROC curve plots the true positive rate versus the false positive rate.

To summarize these curves, the DREAM organizers proposed different statistics:

- AUPR: The area under the PR curve.
- AUROC: The area under the ROC curve.
- AUPR p-value: The probability that a given or larger AUPR is obtained by random ordering of the potential network edges.
- AUROC p-value: The probability that a given or larger AUROC is obtained by random ordering of the potential network edges.

An overall score was used to evaluate the predictions for the five networks of each subchallenge:
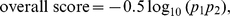
where 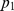 and 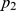 are respectively the geometric means of AUPR p-values and AUROC p-values taken over the five networks.

### Results on the DREAM4 Multifactorial Data

#### Challenge

We took part in the DREAM4 *In Silico Multifactorial* challenge, where the goal was to provide the ranking of the potential (directed) regulatory interactions for five simulated networks. At the time of submission, the gold standard networks were unknown and it was thus impossible to choose the best one among several tree-based methods at our disposal. We thus submitted the rankings obtained by our GENIE3 procedure using the Random Forests algorithm with 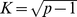.

Among twelve challengers, GENIE3 got the best performance with an overall score of 37.428. As a comparison, the score of the first runner-up was 28.165.

Table 1 shows the AUPR and AUROC values of our predictions and those of the first runner-up, and Table 2 shows their associated p-values, indicating that our predictions were significantly better than random guessing. On all networks, these scores were the highest among the twelve challengers. Individual PR and ROC curves on each network are collected in Figure S1.

**Table 1:** AUPR and AUROC scores for DREAM4 Multifactorial challenge.

	Method	NET1	NET2	NET3	NET4	NET5
AUPR	GENIE3-RF-sqrt	0.154	0.155	0.231	0.208	0.197
	2nd best	0.108	0.147	0.185	0.161	0.111
AUROC	GENIE3-RF-sqrt	0.745	0.733	0.775	0.791	0.798
	2nd best	0.739	0.694	0.748	0.736	0.745

GENIE3-RF-sqrt: GENIE3 using Random Forests with 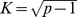. 2nd best: Second best performer in the DREAM4 Multifactorial challenge.

**Table 2:** AUPR and AUROC p-values for DREAM4 Multifactorial challenge.

	Method	NET1	NET2	NET3	NET4	NET5	Overall p-value
AUPR p-value	GENIE3-RF-sqrt	3.3e-34	7.9e-54	1.8e-54	5.5e-47	4.6e-44	1.0e-46
	2nd best	5.6e-23	9.7e-50	6.6e-43	1.5e-35	4.4e-23	7.4e-35
AUROC p-value	GENIE3-RF-sqrt	3.3e-18	1.1e-28	9.7e-34	6.7e-33	1.9e-34	1.4e-29
	2nd best	1.7e-17	5.4e-21	4.9e-28	1.9e-23	1.1e-24	6.3e-23

GENIE3-RF-sqrt: GENIE3 using Random Forests with 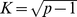. 2nd best: Second best performer in the DREAM4 Multifactorial challenge.

#### Comparison of Tree-based Methods

We have subsequently applied GENIE3 on these same datasets, using the Extra-Trees algorithm, and also setting *K* to its maximum value (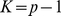). Table 3 shows the overall scores obtained with the four different combinations. The Random Forests and the Extra-Trees algorithms gave comparable results, and the predictions were improved when the parameter *K* was increased, i.e. when the randomization was reduced. The overall best result was achieved when we used Random Forests with 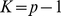, giving an overall score equal to 40.471. This result is slightly better than our initial submission to the challenge. Unless otherwise stated, all subsequent experiments in the paper will be carried out with this particular setting. Note that in this case, the algorithm simply corresponds to the Bagging method applied on standard regression trees [41].

**Table 3:** Overall scores of GENIE3 for DREAM4 networks.

	RF-sqrt	RF-all	ET-sqrt	ET-all
Overall score	37.428	40.471	35.881	40.111

RF: Random Forests, ET: Extra-Trees, sqrt: 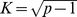, all: 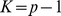.

#### Detailed Analysis of the Predictions

**Figure 2:**
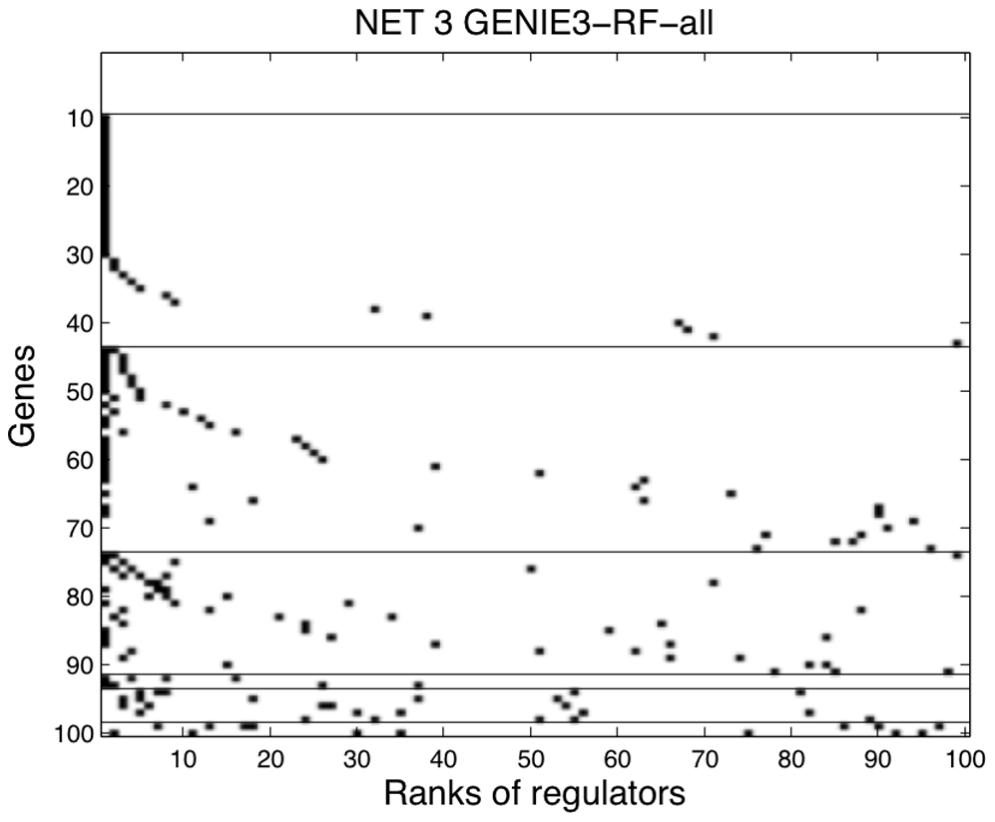
Detailed results on DREAM4 NET3. Ranking of the regulators for all genes. Each row corresponds to a gene. Dots in each row represent the positions in the Random Forests ranking of the regulators of this gene. Genes are ordered on the y-axis according to their number of regulators in the gold standard network; those having the same number of regulators are grouped inside an horizontal block (from no regulator at the top to 6 regulators at the bottom). Inside each block, genes are ordered according to the median rank of their regulators. The ranking of interactions was obtained with Random Forests and 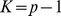.

To have a more precise picture of the quality of the predictions obtained with GENIE3, Figure 2 depicts the ranking of regulators for all genes, grouped according to their number of regulators, for the third network which was predicted with the highest AUPR score by our method. Similar plots for the other networks can be found in Figure S2.

As can be seen from this figure, GENIE3 is able to retrieve the best regulator for about two thirds of the genes that have only one regulator. For genes with two regulators, the method retrieves one of the two regulators for about the same proportion of genes but is less good at retrieving the second regulator (only for one gene, the two regulators are at the top of the ranking). For genes with three or more regulators, even one regulator seems to be difficult to retrieve.

**Figure 3:**
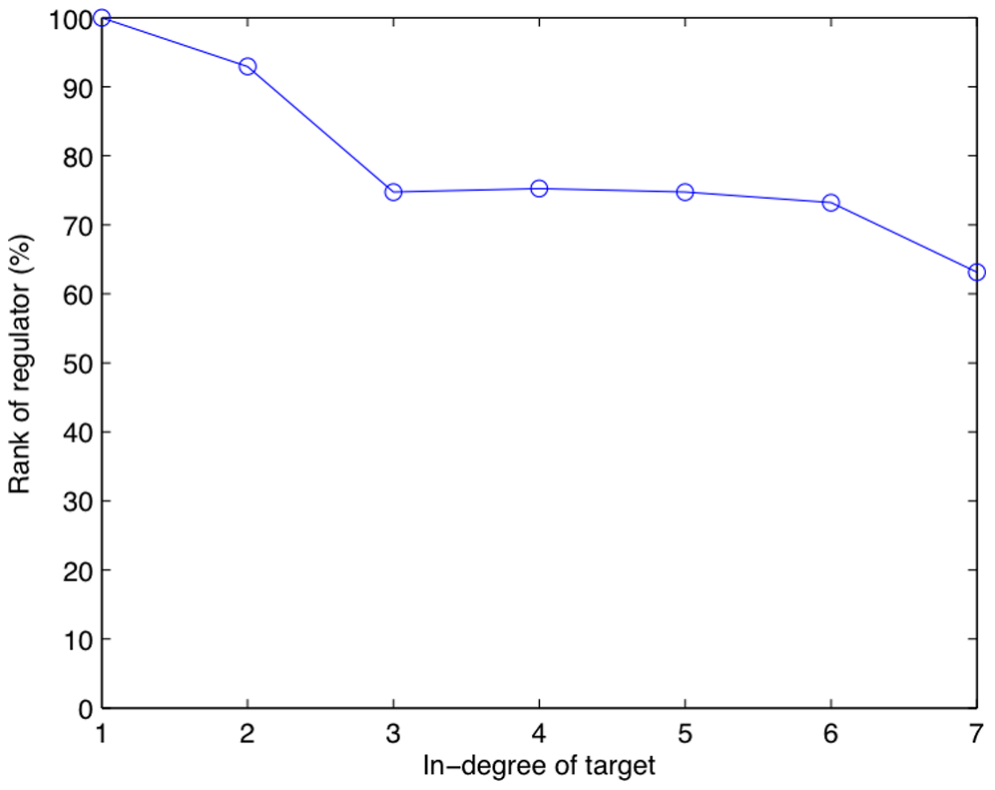
Rank of regulators as a function of the in-degree of the target. The in-degree of a target is its number of regulators. The dot corresponding to in-degree *n* is the median rank of regulators that regulate a gene with in-degree *n*, over the five networks. The rank is presented here as a percentage, such that the first and last regulators of each ranking have a rank equal to 100% and 0% respectively. The ranking of interactions was obtained with Random Forests and 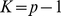.

This suggests that the performance of GENIE3 at retrieving a regulator of one gene degrades as the number of regulators of this gene increases, as also observed from the analysis of the results of the DREAM3 challenge in [42]. To further check this hypothesis, we plotted in Figure 3 the median rank of the regulators of gene *j*, such that gene *j* is regulated by an increasing number of genes. The rank is presented here as a percentage, such that the first and last regulators of the ranking have a rank equal to 100% and 0% respectively. This plot clearly shows that the quality of the ranking monotonically decreases with the in-degree of the genes.

#### Undirected Versus Directed Predictions

One interesting feature of GENIE3 is its potential ability to predict directed networks, while methods based on mutual information or correlation are only able to predict undirected networks.

To see to what extent the networks predicted by our method are asymmetric, we show in Table 4 for each network the proportion of predicted regulatory links for which the opposite link is not predicted. Notice that these predictions were obtained from the Random Forests ranking, by fixing a weight threshold such that the predicted network contains the same total number of edges as the gold standard. This percentage is compared with the same percentage computed for the gold standard. Our predicted networks are clearly more symmetric than the corresponding gold standards but they nevertheless contain a significant number of asymmetric predictions (52% of the links on the average).

**Table 4:** Asymmetry of predicted and gold standard networks.

	NET1	NET2	NET3	NET4	NET5
GENIE3-RF-all	50%	58%	48%	48%	58%
Gold standard	92%	94%	97%	96%	98%

The asymmetry of a network is measured by the proportion of regulatory links for which the opposite link is not predicted.

Of course, the fact that GENIE3 predicts asymmetric networks does not ensure that the prediction of these asymmetric links is really informative; asymmetric predictions might precisely correspond to spurious predictions. To check this, we swapped the weights 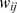 and 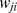 for each pair of genes 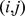 and assessed the new resulting rankings. The overall score dropped from 40.471 to 14.674, suggesting that GENIE3 tends to correctly assign the highest weight to the true direction, given an undirected regulatory link.

To further assess the ability of our method to predict link directions, we computed the proportion of edges 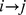 in the gold standard network such that there is no edge 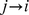 and for which our method wrongly predicts 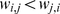. This can be considered as an error rate when our method is used for directing the edges of a known undirected network. Table 5 shows the average value of this error rate over the five networks, for increasing recall values. Given that there are only two choices for a given link, a random ranking of the directed interactions would yield an error rate close to 50%. For all recall values, the error rate is significantly lower than 50% suggesting that our method is a plausible approach for directing an undirected network. The error rate is smaller (20%) for the top ranked interactions but it remains quite good (27%) even when considering less confident predictions.

**Table 5:** Error rates on edge directionality on DREAM4 networks.

Recall	5%	25%	50%	75%	100%
Error rate	20%	28%	27%	27%	26%

The error rate is the proportion of edges 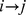 in the gold standard network such that there is no edge 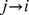 and for which our method wrongly predicts 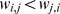. Each column corresponds to one value of the number of considered directed links of the gold standard. These error rates were obtained with Random Forests and 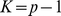, and averaged over the five networks.

Finally, we compared GENIE3 to three existing approaches based on the computation of mutual information (MI), namely CLR [11], ARACNE [12] and MRNET [14], and to one approach based on graphical Gaussian models (GGMs) [20]. All these four methods can only predict undirected networks. For these experiments, we used the original MatLab implementation of CLR [43] and the implementations of ARACNE and MRNET in the minet R package [44]. To compute mutual information, we used a B-spline smoothing and discretization, as implemented in the CLR package, with the parameter setting used in [11] (10 bins and third order B-splines). For ARACNE, the tolerance parameter was optimized between 0 and 15%, as advised in [12]. For GGMs, we used the GeneNet R package [45].

We carried two evaluations, the first one against the undirected gold standard (Table 6) and the second one against the directed gold standard (Table 7). In the first case, the predictions of GENIE3 were symmetrized by assigning to each pair 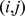 the maximum between 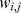 and 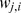. In the second case, links 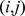 and 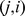 were both assigned the same weights by the four undirected methods, while GENIE3 was used unmodified. In the undirected case, GGMs give the lowest score while all MI-based methods are equally good with only a slight advantage to our method. In the directed case, GENIE3 is significantly better than the four other methods that are constrained to predict undirected links.

**Table 6:** Overall scores for the undirected networks of DREAM4.

	GENIE3-RF-all	CLR	ARACNE	MRNET	GGM
Overall score	36.736	35.838	32.632	34.124	26.846

Links 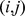 and 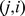 were both assigned the same weights by CLR, ARACNE, MRNET, and GGM, while the predictions of GENIE3 were symmetrized by assigning to each pair 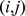 the maximum between 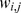 and 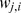.

**Table 7:** Overall scores for the directed networks of DREAM4.

	GENIE3-RF-all	CLR	ARACNE	MRNET	GGM
Overall score	40.471	31.57	28.488	30.435	23.705

Links 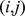 and 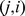 were both assigned the same weights by CLR, ARACNE, MRNET, and GGM, while GENIE3 was used unmodified.

#### Performance on *Escherichia coli* Dataset

**Figure 4:**
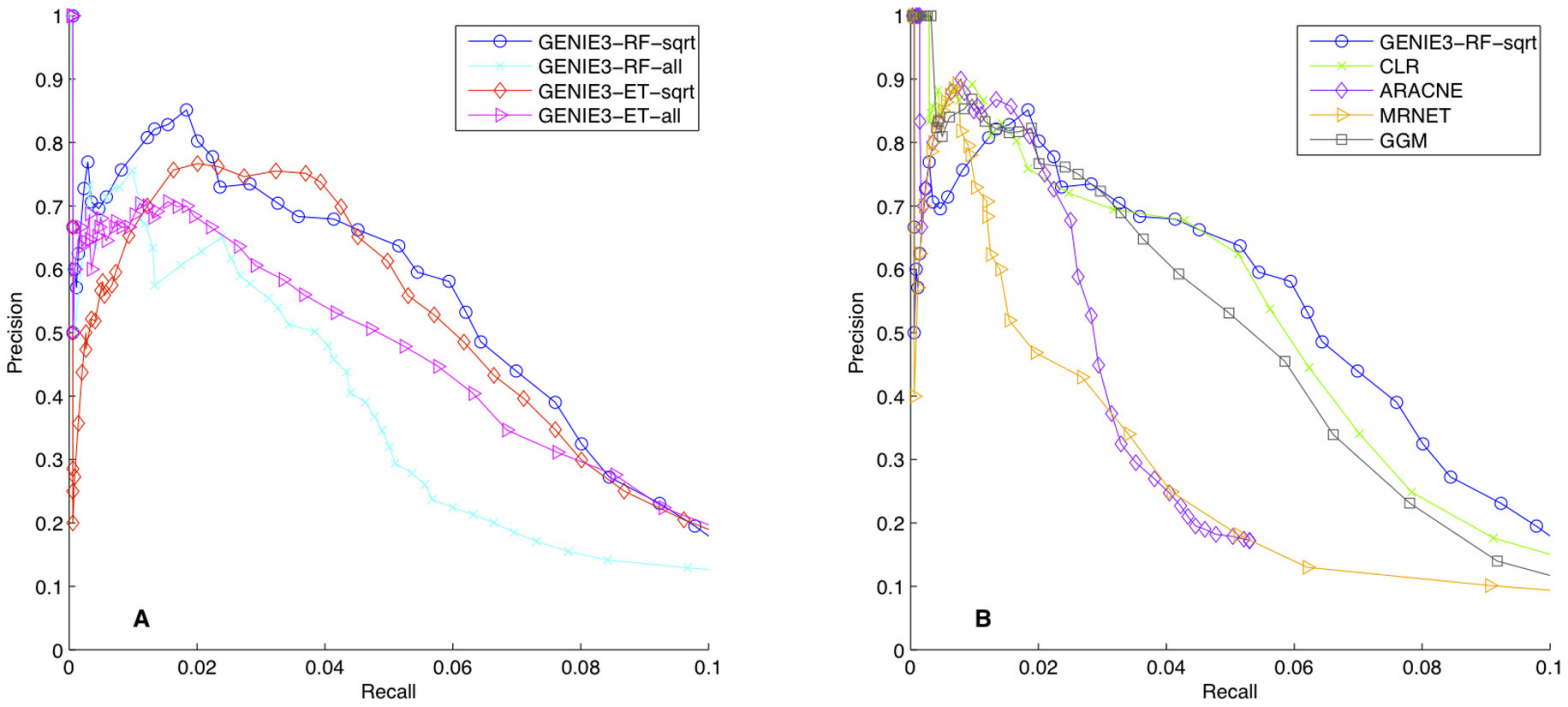
Precision-Recall curves for the *E. coli* network. Only known transcription factors were used as input genes. A. Comparison between the four different settings of the tree procedure. B. Comparison to other approaches.

As a first experiment on the real *E. coli* dataset, we adopted the same evaluation protocol as in [11] that assumes that we have prior knowledge about which genes of the gold standard (i.e. the experimentally confirmed interactions curated in RegulonDB) are transcription factors. In the context of our method, this makes each feature selection problem much easier as the regulators have to be identified among a much smaller set of genes. This also makes undirected and directed methods equally applicable (since all links are automatically directed from transcription factors to genes). Figure 4A shows the precision-recall curves for the four different settings of the tree-based procedure. Contrary to the DREAM4 networks, setting 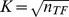, where 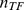 is the number of potential regulators, improves the performance compared to 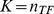, RF-sqrt leading to the best precision-recall curve. Figure 4B compares this method with the four undirected methods, CLR, ARACNE, MRNET, and GGMs, using exactly the same protocol. The predictions obtained using GENIE3 with Random Forests and 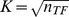 outperform those obtained from ARACNE and MRNET, and give a precision-recall curve comparable with CLR and GGMs (although less good for small recall values). Figure S3 shows the ranking of the regulators with GENIE3-RF-sqrt for all genes grouped according to their number of regulators.

As a second experiment, we simulated conditions similar to the DREAM4 challenge, where transcription factors were unknown and tried to infer the network using as input features in each step of our procedure all 1471 genes except the target gene itself. For this experiment, precision never exceeded 6%, even for the smallest values of recall. This indicates that the predictions are extremely poor and only slightly better than random guessing.

With respect to the results obtained in the DREAM4 challenge, these results are disappointing. The larger number of genes in this case does not explain everything since it also comes with an increase of the number of observations. Actually in both cases, the number of observations is comparable to the number of genes. However, since the *E. coli* dataset is a collection of expression data compiled from experiments carried out in different laboratories, there may be some redundancy among these experiments or some bias in their selection. They are thus probably not as statistically useful as the really randomized and i.i.d. perturbation data generated for the DREAM4 multifactorial challenge. Other potential reasons for these poor results are the fact that the gold standard network is not complete and also the discrepancy that exists between the simulation model used to generate the DREAM4 data and the real regulation mechanism of *E. coli*.

## Discussion

We developed GENIE3, a procedure that aims at recovering a gene regulatory network from multifactorial expression data. This procedure decomposes the problem of inferring a network of size *p* into *p* different feature selection problems, where the goal is to identify the regulators of one of the genes of the network. Among different feature selection methods, we chose to use tree-based ensemble methods. These methods do not make any assumption about the nature of gene regulation, can potentially deal with combinatorial regulations and non-linearity. They work well in the presence of a large number of genes, are fast to compute and scalable.

GENIE3 got the best performance on the DREAM4 *In Silico Multifactorial* challenge and is competitive with existing algorithms to decipher the genetic regulatory network of *Escherichia coli* assuming that transcription factors are known. When no prior knowledge is available about transcription factors, our results on the *E. coli* network were however not better than random guessing. The reason of this discrepancy with respect to the results on the DREAM4 challenge deserves to be further analysed.

Our algorithm can be improved along several directions. As tree-based ensemble methods, we used the Random Forests and the Extra-Trees algorithms, that both gave comparable results. However, the performances of these methods depend to some extent on their main parameter, the number *K* of randomly selected attributes at each node of one tree. On the DREAM4 *Multifactorial* datasets, improved predictions were obtained by increasing *K* to its maximum value (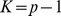), while on the *E. coli* dataset, the best ranking of interactions was obtained by using 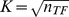. It would thus be of interest to find a way to automatically tune this parameter. A first solution could be to select the value of the parameter that leads to the best performance for the prediction of the expression values, i.e. that minimizes mean square error in (2) estimated by cross-validation. Unfortunately, this solution did not work on the *E. coli* dataset, where using 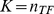 led to lower mean square error but a less good precision-recall curve.

There is also a potential room for improvement on the way variable importance scores are normalized. One apparent drawback of the measure we proposed is that it does not take into account the quality of the trees in generalization. Indeed since our trees are fully grown, importance weights satisfy equation (4) which, given our normalization, attributes equal weights to all tree models irrespective of their quality when used to predict the expression values of the target gene. We tried to correct for this bias by normalizing the variable importance scores by the effective variance reduction brought by the model as estimated by cross-validation but it actually deteriorated the performances. The question of the optimal normalization remains thus open at this stage.

In this paper, we focused on providing a ranking of the regulatory interactions. In some practical applications however, one would like to determine a threshold on this ranking to obtain a practical predicted network. To address this question, we have tried to exploit cross-validation estimates of the mean-square error as a criterion to determine such a threshold but we have not been successfull so far. As future work, we therefore would like to extend the technique developed in [46] to better assess the significance of the predicted regulatory links and thus help determining a threshold.

Our experiments on the DREAM4 dataset show that GENIE3 is able to predict the direction of the edges to some extent, even though it only exploits steady-state measurements. This is an interesting result as this is commonly admitted to be a difficult problem. Bayesian networks also potentially allow to predict edge directionality. A comparison with this family of methods would be an interesting future work direction. Note that with respect to our approach, Bayesian networks do not allow for the presence of cycles in the predicted network, which could be a limiting factor for networks such as those in DREAM4 that contain cycles by construction.

Several procedures using regression trees have already been proposed to solve the regulatory network inference problem. Most of these procedures exploits other kinds of data in addition to expression data, e.g. counts of regulatory motifs that serve as binding sites for transcription factors [47], [48], or ChIP-based binding data [49]. The closest work to ours is the procedure developed by Segal et al. [50], that recovers module networks from expression data, so that the genes in each module share the same regulators in the network and the same conditional probability distribution, represented by a (single) regression tree.

Finally, although we exploited tree-based ensemble methods, our framework is general and other feature selection techniques could have been used as well. Actually, several existing methods for network inference can be interpreted as special instances of this framework. In particular, mutual information as used in Relevance Networks [10] or CLR [11] is a common dependency measure exploited in filter-kind approaches for feature selection [28]. MRNET [14] also considers each gene in turn as the target output and exploits the maximum relevance/minimum redundancy feature selection method to rank its candidate regulators. Like Relevance Networks and CLR, this method reduces all the information contained in the expression data to mutual information between all pairs of genes, while our approach is by nature multivariate. Meinshausen and Bühlmann [21] show that finding the zero entries in the inverse covariance matrix of a multivariate Gaussian distribution can be solved by applying the LASSO embedded feature selection mechanism using each gene in turn as the target output, which links Gaussian graphical models with our approach. While the latter assumes that the functions 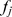 in (1) are linear, our approach can be seen as a relaxation of this assumption by exploiting a non-parametric supervised learning method. Whether or not this is an advantage in practice for inferring regulatory networks is still an open question that deserves to be studied.

### Software Availability

Our GENIE3 software is available from http://www.montefiore.ulg.ac.be/~huynh-thu/software.html.

## Supporting Information

Figure S1 PR and ROC curves for each DREAM4 Multifactorial network. Left: PR curves. Right: ROC curves. Prec: Precision. FPR: False Positive Rate. TPR: True Positive Rate. The rankings of interactions were obtained using Random Forests and *K* = √*p*−1.
(3.04 MB TIF)

Figure S2 Ranking of the regulators for all genes on DREAM4 networks. Each row in a figure corresponds to a gene. Dots in each row represent the positions in the Random Forests ranking of the regulators of this gene. Genes are ordered on the y-axis according to their number of regulators in the gold standard network; those having the same number of regulators are grouped inside an horizontal block. Inside each block, genes are ordered according to the median rank of their regulators. The rankings of interactions were obtained with Random Forests and *K* = *p*−1.
(8.11 MB TIF)

Figure S3 Ranking of the regulators for all genes on the *E. coli* network. Each row in a figure corresponds to a gene. Dots in each row represent the positions in the Random Forests ranking of the regulators of this gene. Genes are ordered on the y-axis according to their number of regulators in the gold standard network; those having the same number of regulators are grouped inside an horizontal block. Inside each block, genes are ordered according to the median rank of their regulators. Only known transcription factors where used as input genes. The ranking of interactions was obtained with Random Forests and *K* = √ *n_TF_*.
(3.06 MB TIF)
