# Supplementary figures and images for: Inferring Regulatory Networks from Expression Data Using Tree-Based Methods
Source: PLoS One. 2010 Sep 28;5(9):e12776. doi: 10.1371/journal.pone.0012776 (PMC2946910; doi:10.1371/journal.pone.0012776)

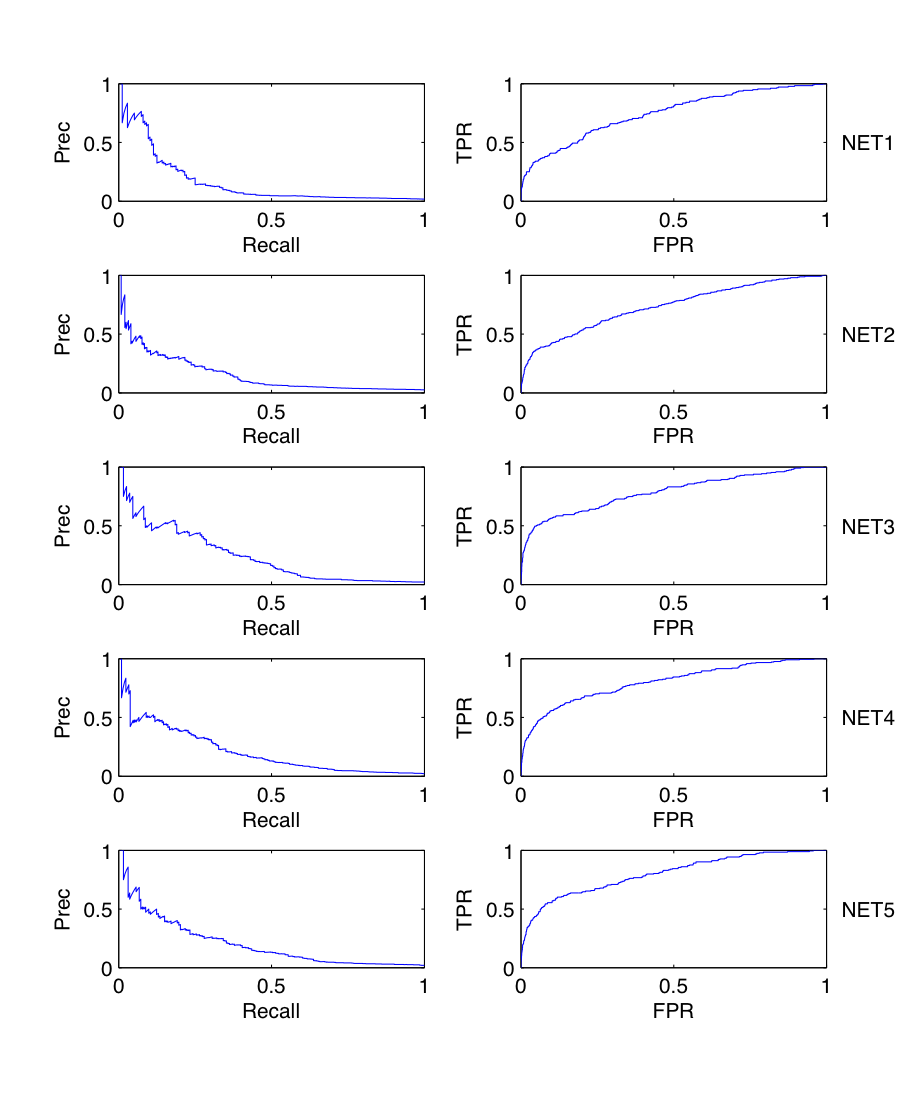

Supplement: Figure S1 — PR and ROC curves for each DREAM4 Multifactorial network. Left: PR curves. Right: ROC curves. Prec: Precision. FPR: False Positive Rate. TPR: True Positive Rate. The rankings of interactions were obtained using Random Forests and K = √p−1. (3.04 MB TIF) [file pone.0012776.s001.tif]

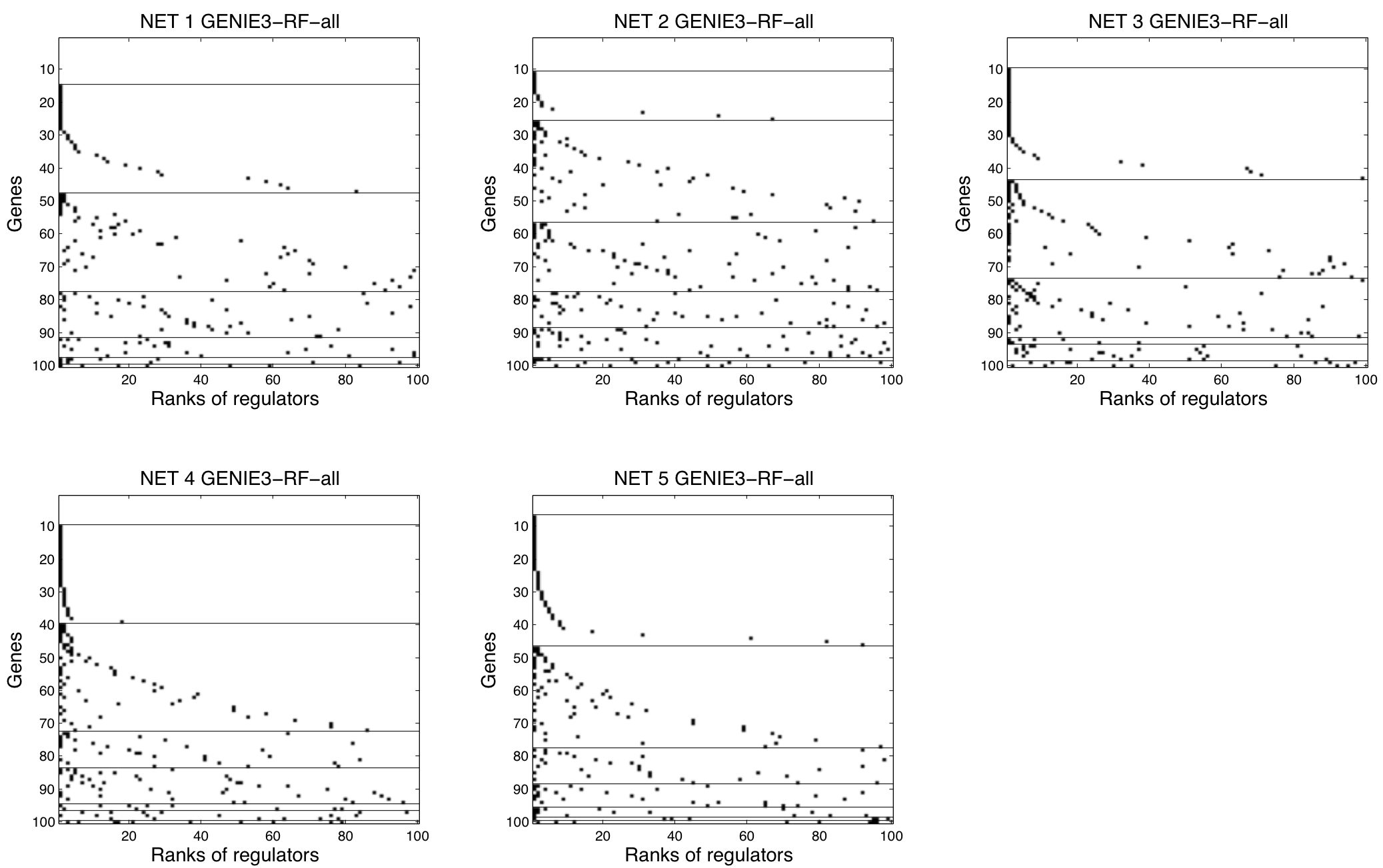

Supplement: Figure S2 — Ranking of the regulators for all genes on DREAM4 networks. Each row in a figure corresponds to a gene. Dots in each row represent the positions in the Random Forests ranking of the regulators of this gene. Genes are ordered on the y-axis according to their number of regulators in the gold standard network; those having the same number of regulators are grouped inside an horizontal block. Inside each block, genes are ordered according to the median rank of their regulators. The rankings of interactions were obtained with Random Forests and K = p−1. (8.11 MB TIF) [file pone.0012776.s002.tif]

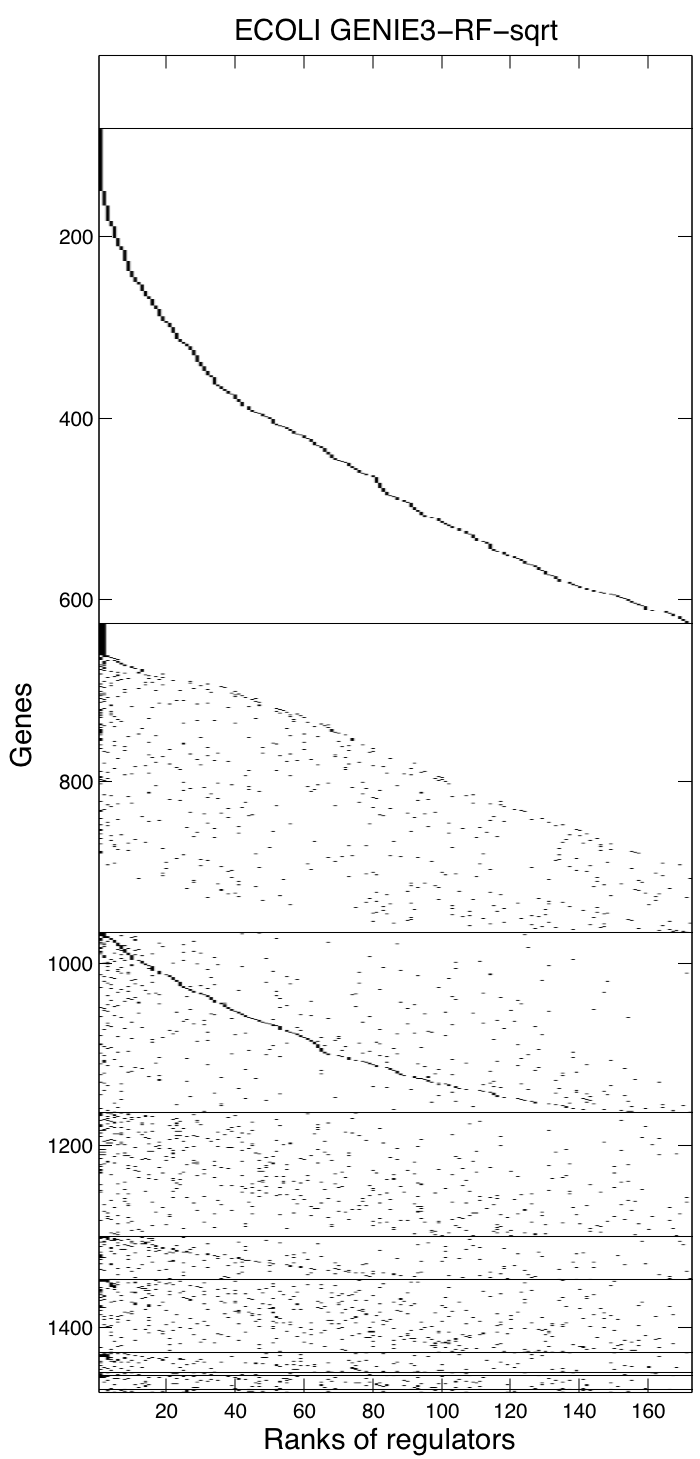

Supplement: Figure S3 — Ranking of the regulators for all genes on the E. coli network. Each row in a figure corresponds to a gene. Dots in each row represent the positions in the Random Forests ranking of the regulators of this gene. Genes are ordered on the y-axis according to their number of regulators in the gold standard network; those having the same number of regulators are grouped inside an horizontal block. Inside each block, genes are ordered according to the median rank of their regulators. Only known transcription factors where used as input genes. The ranking of interactions was obtained with Random Forests and K = √ nTF. (3.06 MB TIF) [file pone.0012776.s003.tif]
